# The role of RAC1 in resistance to targeted therapies in cancer

**DOI:** 10.1080/21541248.2025.2505977

**Published:** 2025-05-21

**Authors:** Cristina Uribe-Alvarez, Jonathan Chernoff

**Affiliations:** Cancer Signaling & Microenvironment Program, Fox Chase Cancer Center, Philadelphia, PA, USA

**Keywords:** Signal transduction, cancer, RHO GTPases, RAC1, drug resistance

## Abstract

RAC1 is a small 21 kDa RHO GTPase that plays a pivotal role in regulating actin cytoskeletal dynamics and cell growth. Alterations in the activity of RAC1 are implicated in a range of diseases, including cancer. Increased RAC1 activity, due to overexpression and/or activating mutations, drives transcriptional upregulation, reactive oxygen species production, mesenchymal-to-epithelial transition, membrane ruffling, and uncontrolled cell proliferation, which are hallmarks of an oncogenic phenotype. While RAC1-activating mutations alone do not appear sufficient to transform cells, their combination with other common mutations, such as BRAF, NRAS, or NF1, have been linked to drug resistance and significantly worsen patient prognosis and hinder treatment responses. The precise mechanisms underlying drug resistance, and the regulation of *RAC1* splicing remain poorly understood. RAC1 is a challenging therapeutic target due to its ubiquitous presence and essential cellular functions. To date, there are no established standard treatments for cancers that harbour an additional RAC1 mutation or for RAC1-mediated drug resistance. Current experimental strategies aim to target RAC1 localization, its activators (*e.g*. guanine nucleotide exchange factors) and downstream effectors. Regulating RAC1 expression by targeting epigenetic regulators, and direct targeting of RAC1 itself, may also be possible in the near future.

Small GTPases (20–30 kDa) are hydrolases that function as binary on-and-off switches that cycle between an active guanosine triphosphate (GTP)-bound state and an inactive guanosine diphosphate (GDP)-bound state [1]. These low molecular weight proteins (20–30 kDa) regulate a vast number of cellular processes involved in cell proliferation, survival, metabolism, and motility [2–5]. Small GTPases can be grouped according to their sequence and function into five major families: RAS, RHO, ARF, RAN and RAB [6]. The RHO GTPase subfamily – subdivided into seven subfamilies: RHO, RAC, CDC42, RND, RHOD, RHOBTB, and RHOH – is best known for its role in actin dynamics and cell polarity [3]. The RAC GTPase subfamily consists of four members, RHOG and the Ras-Related C3 Botulinum Toxin Substrates RAC1, RAC2, RAC3 [3,7].

The *RAC1* gene is localized on chromosome 7 (7p22.1) and it encodes a 192 amino acid, 21 kDa, plasma membrane-associated protein [1]. The GC-rich *RAC1* promoter is localized 113–30 bp upstream of *RAC1* transcription site and like typical housekeeping genes, it does not have a TATA- and a CCAAT-box [1,8]. *RAC1* contains seven exons, that can produce two proteins through alternative splicing: RAC1 (exons 1–6), and the splicing variant RAC1B that includes the expression of an additional exon 3B (exons 1–3, 3b, 4–6) (Figure 1(a)) [11]. RAC1 structure contains two mobile regulatory switch domains, Switch I (residues 25 to 39), and Switch II (residues 57 to 75) that undergo conformational changes during the exchange of GDP/GTP and allow RAC1 to interact with its effector proteins and activate downstream signalling molecules [12]. Like the RAS superfamily, RAC1 has a highly conserved central guanine nucleotide-binding domain pocket that includes a) the phosphate-binding sequences GDGAVGKT (residues 10–17) and DTAG (residues 57–60), and b) the guanine recognition loop TK(L/K)D (residues 114–117) and SAL (residues 158–160) [13,14]. RAC1’s globular structure folds into a compact six strand-β-sheets and six α-helix protein (Figure 1(a)).

**Figure 1.**
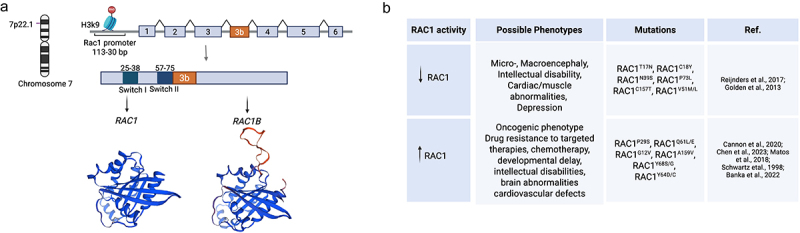
RAC1 mutations and their association with diverse diseases. A) *RAC1*, localized on chromosome 7 (7p22.1), encodes a 192 amino acid 21 kDa protein. The *RAC1* promoter is localized 113–30 bp upstream the transcription initiation site. *RAC1* has 7 exons that can produce two proteins: RAC1 (exons 1–6) and the fast-cycling isoform RAC1B (exons 1–3, 3b, 4–6). Exon 3b adds a 19 amino acid in-frame insertion after the switch-II domain (orange) that induces an open switch I conformation and a highly mobile switch II. RAC1s tertiary structure consists of a 6 strand β-sheet domain surrounded by six α helices. RAC1 (PDB-3TH5) [9], and RAC1B (PDB-1RYF) [10] protein models were generated using swissmodel.Expasy.org B) RAC1 is tightly regulated: decreased RAC1 signalling is related to neurodegenerative disorders, mental retardation and micro- and macrocephaly, epilepsy and cardiovascular diseases; while increased activity of RAC1 can accelerate cells oncogenic phenotype, confer drug resistance tumours and worsen patients prognosis. Created in BioRender. Uribe, C. (2025) https://bioRender.com/e76i718

RAC1 activity is tightly controlled by growth factors, phospholipids, integrins, and upstream regulators [15]. RAC1 activity can be decreased by GTPase-activating proteins (GAPs), which accelerate hydrolysis of GTP to GDP, resulting in a decrease of active RAC1-GTP; or by Guanine-nucleotide dissociation inhibitors (GDIs), which (a) sequester inactive RAC1 in the cytoplasm, preventing it from exchanging GDP to GTP; (b) interact with RAC1-GTP and inhibit GTP hydrolysis and its interaction with effector proteins; (c) and bury the hydrophobic prenyl group of RAC1 within a hydrophobic groove of its immunoglobulin–like domain, sequestering RAC1 in a cytosolic soluble pool and preventing it from anchoring to the plasma membrane [16]. GDIs also restrict RHO GTPases access to activating guanine-nucleotide exchange factors (GEFs) and GTPase activating proteins (GAPs) [17]. GDIs have a phosphorylation-mediated release mechanism that controls and coordinates RHO GTPase activation. Phosphorylation of the RAC1–RHOGDIα complex by PAK1 (p21-activated kinase 1) or by the calcium-dependent PKCα (Protein kinase C α) decreases the stability of the complex and induces conformational changes that release RAC1 and allow it to be further activated or transported [17,18].

RAC1 requires the help of guanine-nucleotide exchange factors (GEFs) to exchange GDP for GTP. The binding of a GEF to RAC1 induces conformational changes in the switch regions and the phosphate binding area that stimulate the release of GDP. Then the GEF is displaced by the subsequent binding of a GTP molecule [19]. There are ~85 GEFs capable of promoting GDP/GTP exchange activity on RHO GTPases, and about half can act on RAC isoforms (Table 1). Most RAC-GEFs belong to the Diffuse B-cell lymphoma (DBL)-like class, which has a prototypical N-terminal DH (DBL homology) domain responsible for GDP/GTP exchange activity, often in tandem with a PH (pleckstrin homology) domain that acts as binding sites from PI3K lipid products [62]. Mutated or overexpressed GEFs can elevate the rate of GDP/GTP exchange of RAC1 resulting in oncogenic phenotypes that include increased cell invasion, drug resistance and metastasis [63,64].

**Table 1.** RAC1 nucleotide exchange factors (GEF) specificity.

GEF	Specificity	Reference
KALIRIN 5/7	RAC	[20]
TIAM1/TIAM2	RAC	[21–24]
SOLO/TRIO8	RAC	[20]
DOCK1/2	RAC	[25–28]
P-REX2	RAC	[29]
b-PIX/COOL1	RAC/CDC42	[27,30,31]
FGD5	RAC/CDC42	[28,32]
PLEKHG1/G2/G3	RAC/CDC42	[33–35]
ASEF1/2	RAC/CDC42	[36,37]
DOCK6/7/10	RAC/CDC42	[38]
VAV1/2/3	RAC/CDC42	[39,40]
ARHGEF39	RAC/RHO	[23]
KALIRIN 12	RAC/RHO	[20]
TRIO FL	RAC/RHO	[20]
Ephexin1/3/4/5	RAC/CDC42/RHO	[41–43]
DEF6	RAC/CDC42/RHO	[44–46]
P-REX1	RAC/CDC42/RHO	[47–51]
a-PIX/COOL2	RAC/CDC42/RHO	[30,52]
FARP1/FERM	RAC/CDC42/RHO	[23,53,54]
GEFT (p63RhoGEF)	RAC/CDC42/RHO	[55]
ARHGEF16	RAC/CDC42/RHO	[43]
Ras-GRF1/2	RAC/RAS	[56–59]
SOS1/2	RAC/RAS	[60,61]

Cytosolic RAC1 can be mobilized to the plasma membrane, where it regulates actin dynamics and membrane protrusions, thereby controlling cell motility and adhesion [65,66], or it can migrate to the nucleus, where it regulates nuclear membrane organization, enhances cell cycle progression, division, and increases transcriptional activation of Wnt target genes [67–71]. RAC1 subcellular localization unbalance and abnormal nuclear accumulation has been linked to cancer progression and invasion [71–73]. Cells with nuclear-export deficiencies (including the knockdown of the B23 nuclear chaperone protein that exports RAC1 from the nucleus to the cytoplasm) accumulate RAC1 in the nucleus, creating an unbalance that increases RHO/ROCK signalling and results in increased cell proliferation and migration [73]. RAC1 accumulation in the nuclei has been found in PC3, LM4175 lung carcinoma and OCI-AML3 acute myeloid leukaemia cells lines. Additionally, RAC1 nuclear localization is also predominantly detected in prostate cancer tissues compared to benign tissues [73]. In breast cancer, RAC1 can also interact with oestrogen receptors α (ERα) that are predominantly found in the cell’s nucleus. RAC1 and ERα protein levels appear to be positively correlated, with RAC1 modulating transcriptional activity of ERα [74]. RAC1 targeting has shown to reduce ER transcriptional activity and cell proliferation on ER positive breast cancer [74,75].

RAC1 is a key signalling molecule that is ubiquitously expressed in human tissues [14,76]. RAC1 regulates various downstream effector proteins that contribute to epithelial differentiation, cellular motility, cytoskeletal reorganization, cell-to-cell adhesion, proliferation, cell cycle, phagocytosis, NADPH, ROS activity, and inflammatory responses [77]. RAC1 integrates cellular signals to control actin dynamics and migration of various immune cells, including neutrophils, B cells and macrophages influencing their function and distribution during inflammation [78,79]. RAC1 also reorganizes neuron cytoskeletons contributing to dendritic spine formation, maintenance, morphogenesis and synaptic plasticity [80]. In adipose tissue and skeletal muscle, RAC1 participates in insulin response, facilitating the translocation of GLUT4 glucose transporter vesicles to the plasma membrane, thereby allowing glucose entry into cells and decreasing blood glucose [81,82]. RAC1 forms part of the NAPDH oxidase complex that participates in oxidative metabolism and reactive oxygen production and can further influence mitochondrial function and inflammatory processes [83–85]. Therefore, anomalous or dysfunctional RAC1 signalling is implicated in various human pathologic conditions, including neurodegenerative disorders, mental retardation and micro-and macrocephaly, epilepsy, cardiovascular diseases, diabetes and cancer [6,8,85–91]. *RAC1* missense mutations tend to cause developmental delay and brain malformations, while *RAC1* deletion in mice and drosophila leads to embryonic lethality [90,92,93]. The dominant negative mutant RAC1^T17N^ affinity for GTP is 10 times lower than for GDP, and is predominantly found in either a nucleotide-free state or bound to GDP in an inactive state [94]. Unlike RAC1, RAC1^T17N^ in not regulated by RhoGDIs. However, it can strongly associate with GEFs, thereby competing and reducing wild-type RAC1 activation [95]. Furthermore, RAC1^T17N^ downstream signalling appears to be interrupted since it fails to interact with key effectors such as PAK1 [63,95–97]. In drosophila, expression of RAC1^T17N^ disrupts fusion and morphology in drosophila myoblasts affecting muscle formation [93] (Figure 1(b)).

## RAC1 mutations in cancer

In human cancers, gain of function mutations in *RAC1* result in increased RAC1 signalling which contributes to cancer cell growth and invasion of local and distant tissues. *RAC1* mutations promote tumorigenesis in melanoma, non-small cell lung cancer, breast cancer, prostate cancer, colorectal cancer, head and neck cancer, testicular germ cell tumours, glioblastoma multiforme, hepatocellular carcinoma and thyroid carcinoma among others [11,78,86,98–104]. Activated RAC1 mutations accelerate tumour growth, induces epithelial–mesenchymal transition (EMT), increases angiogenesis, and influences tumour microenvironment-mediated immune escape driving tumour metastasis and causing treatment failures [66,86,98,105–108]. Both *in vitro* and *in vivo* studies indicate that wild-type RAC1 has an essential role in RAS-mediated transformation [109], and RAC1 overexpression is linked to worse prognosis in cancer patients [78,110–112]. Activated forms of RAC1 influence the overall survival and drug resistance in different types of cancer, and RAC1 expression levels have been proposed as a prognostic biomarker for patient survival [98,108,111,113–116]. RAC1 gain-of-function mutations have been reported to increase tumours resistance to chemotherapy, chemo-radiation, targeted therapy and anti-hormonal therapy [105,106]. Such mutations also result in resistance to targeted therapy BRAK and MEK drugs in melanoma [108,111,115], resistance to cisplatin in oesophageal squamous cell carcinoma [117] and to irradiation therapies in lung cancer [118]. RAC1^Q61L^ and RAC1^G12V^ are constitutively active forms of RAC1 that are locked in the GTP-bound active state and are unaffected by GAPs and GDIs [17,93,119,120]. Other mutations such as RAC1^P29S^ and RAC1^A159V^ are fast cycling mutations in which, unlike constitutively activating mutations, the intrinsic GTP hydrolysis ability is still influenced by GDIs. RAC1^A159V^ most commonly occurs in head and neck cancer, while RAC1^P29S^ is a known hotspot mutation in malignant melanoma [12,86,99]. RAC1 activating mutations alone are insufficient to initiate carcinogenesis in skin, colorectal and lung cancer, but cooperation with *BRAF* or *KRAS* mutations results in enhanced proliferation and accelerated tumour growth [9,87,101,104,121].

## RAC1B

The *RAC1* gene has an additional exon 3b that is usually not transcribed. This exon comprises 57 nucleotides which, through alternative RNA splicing generate a fast-cycling form of RAC1 known as RAC1B [98,122,123]. This 19-amino acid in-frame is inserted after the switch-II domain and induces an open switch I conformation, a highly mobile switch II, and creates two potential threonine phosphorylation sites (Figure 1(a)) [1,11]. RAC1B is also regulated by GAPs and GEFs, but cannot be sequestered in the cytoplasm by GDIs, consequently, most RAC1B will remain active [10,12,15]. RAC1B expression keeps RAC1 signalling pathways activated, including the tumour-associated signalling pathways, and enhances transcription, cell–cell adhesion, cell invasiveness and motility [124]. RAC1B is almost exclusively expressed in tumour tissue and is associated with worse overall survival and drug resistance in cancer patients [10,11,98,103,113,114,121,125–128].

*RAC1* alternative splicing regulation is poorly understood; however, a recent publication implicated a long noncoding RNA (LncH19) in this process [129]. In colorectal cancer cells, Cordaro et al. showed that LncH19 is required for RAC1B expression through its association with RBFOX2 and hnRNPM splicing factors. Consistently, CRC patient biopsy samples showed a positive correlation between lncH19 and RAC1B expression [129]. In CRC, RAC1B can also migrate to the nucleus, where it associates with the β-catenin-TCF complex and increases expression of cyclins and pro-tumour signalling [130]. Besides being a fast-cycling isoform of RAC1, it appears that RAC1B structural modifications protect it from proteasomal degradation. The HACE1 E3 ubiquitin ligase is a tumour suppressor that ubiquinates active RAC1 at the Lys147 residue, leading to its degradation. The loss of HACE1 leads to an increase of RAC1 activity that contributes to tumour growth, cell migration and cancer progression [84,131–133]. Unlike RAC1^WT^ and RAC1^L61^, RAC1B HACE1-mediated polyubiquitination is minimal, so the protein does not rapidly degrade, and its pro-tumorigenic effects persist [131,134]. Additionally, when compared to RAC1^WT^ and RAC1 point mutations, RAC1B shows a diminished ability to signal through PAK1, AKT1, and Jun N-terminal kinase (JNK) [127], which is also a critical regulator of RAC1 ubiquitination [119,127,134].

RAC1B expression has been found in skin and epithelial tissues from the intestinal tract, breast carcinoma, HCC, lung cancer, NSCLC, pancreatic adenocarcinoma, thyroid cancers and papillary thyroid carcinomas [1,10,11,98,122,126,128]. RAC1B overexpression synergizes with activating *BRAF* and *KRAS* mutations, worsening the prognosis in follicular thyroid carcinomas, colorectal cancer, and lung adenocarcinomas [101,103,104,121]. RAC1B is presumed to be responsible for chemoresistance to doxorubicin and trastuzumab, tumour recurrence and metastasis in breast cancer stem cells (BCSCs). RAC1B silencing in BCSCs reverted tumours sensitivity to doxorubicin [98]. RAC1B overexpression is also linked to CRC resistance to cetuximab and oxaliplatin, and RAC1B knockdown re-sensitizes tumours to oxaliplatin treatment *in vivo* [113,114]. For these reasons, RAC1B expression may serve as a specific molecular target to re-sensitize chemo-resistant tumours and as a prognostic marker to predict patients’ outcomes and treatment response [98,103,135].

## RAC1^P29S^ and its role in mediating drug resistance

**Figure 2.**
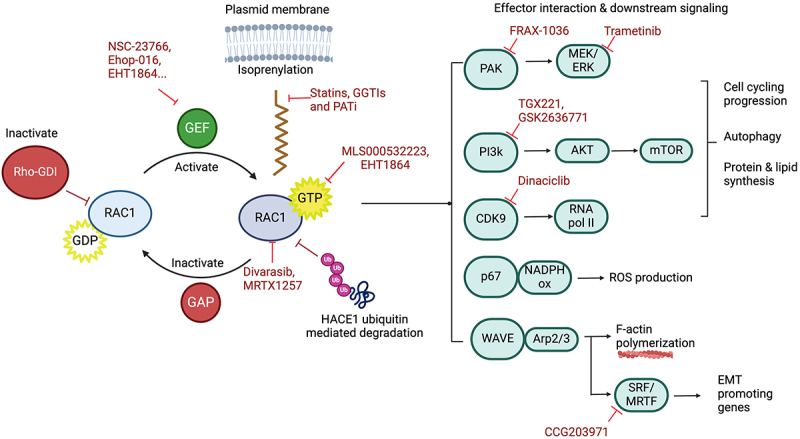
Targeting RAC1 regulation and downstream effectors. RAC1’s GDP-to-GTP exchange is stimulated by guanine-nucleotide exchange factors (GEFs) and inhibited by GTPase activating proteins (GAPs) which accelerate the intrinsic hydrolysis of GTP to GDP; or by GDP-dissociation inhibitors (GDIs) that sequester RAC1 in the cytoplasm. Drugs against RAC1 target its localization, its guanine nucleotide effectors (GEFs), interact directly with RAC1 or RAC1-GTP, or target RAC1 downstream effectors. Created in BioRender. Uribe, C. (2025) https://BioRender.com/e76i718.

*RAC1* mutations are present in 2–9% melanomas, usually in association with activation mutations in *BRAF* or *NRAS*, or inactivating mutations in *NF1* [9,87,111]. The overwhelming majority of *RAC1* mutations in melanoma consist in a change from proline to serine (c.85C > T) in position 29 [9,87,111]. RAC1^P29S^ is the third most common protein-coding hotspot mutation in melanoma after BRAF^V600E^ and NRAS^Q61R/L/K^. The P29S mutation is located within switch I and results in an increased protein flexibility that facilitates GTP interaction and increases nucleotide hydrolyzation [12]. A direct consequence of faster cycling between GDP and GTP is the increase in interaction with effector proteins and cell signalling through Group A PAKs, phosphatidyl inositol-3 kinase (PI3K)-β, and serum response factor (SRF)/myocardin-related transcription factor (MRTF) (Figure 2) [86,136,137]. *RAC1* mutations are insufficient to drive melanoma and are almost always found with other activating MAP kinase hot spot mutations, most frequently *NRAS* (47%), *BRAF* [9,87,111], or *NF1* [9,108,111,138]. Like RAC1B, RAC1^*P29S*^ mutations synergize with *BRAF* and *NRAS* mutations activating the MAPK signalling pathway conferring resistance to targeted therapy drugs such as vemurafenib and dabrafenib monotherapies [108,111,115,116]. In addition, RAC1^*P29S*^ melanoma can evade immune surveillance by over expressing programmed cell death ligand-1 [139].

Interestingly, the RAC1^P29S^ protein, while conferring primary resistance to MEK inhibitors, renders melanoma cells vulnerable to CDK9 inhibitors such as dinaciclib [140]. RNA and protein profiles showed that RAC1^P29S^ expression is associated with elevated activity in signalling enzymes that regulate the G2/M transition, including the cell-cycle activating kinases such as Aurora-A, Polo1-like kinase (PLK)1, WEE1, and CDK9 [140]. Cannon *et al. 2023* recently showed that, in a syngeneic, *in vivo* setting, expressing RAC1^P29S^ in YUMM1.7 *(Braf*^*V600E*^, *Pten*^*null*^, *Cdkn2d*^*null*^) murine melanoma cells, rendered them exquisitely sensitive to dinaciclib especially when combined with a checkpoint inhibitor (anti-PD1). This enhanced response is likely to be related to the upregulation of PD-L1 in cell lines with RAC1^P29S^ mutation [139]. RAC1^P29S^ status in melanoma may therefore represent an important predictive biomarker for both response to targeted drugs against BRAF and MEK resistance and to ICIs [111].

## Epigenetic regulation of RAC1 in cancer and brain disorders

Environmentally induced changes in gene expression can shape an individual’s susceptibility to diseases. DNA methylation, histone methylation/acetylation, and non-coding RNA regulation of a specific chromatin region can lead to abnormal cell growth, cell death evasion, and metastasis [141], and identifying chromatin modulators that sustain tumour growth has emerged as a promising approach to treat cancer. Epigenetic regulation of *RAC1* appears to be implicated in metabolic diseases such as diabetes [8,88]; and cancer [2,5,142–144] and psychiatric disorders, such as depression and substance abuse [8,145,146].

The G9a is a histone methyl transferase is overexpressed in many cancers and influences migration and invasiveness [144,147–149]. G9a knockdown (shG9a) or activity inhibition in alveolar rhabdomyosarcoma (ARMS) upregulated PTEN, reduced phosphorylation of AKT, and decreased RAC1 activity, resulting in a decrease in cell proliferation and defective cell migration. shG9a tumour cells transfected with a RAC1^Q61L^ (a constitutively active RAC1) expressing vector restored the oncogenic phenotype, suggesting that RAC1 is a key downstream effector of G9a [144].

*RAC1* epigenetic alterations modulate behaviours across a range of cellular process and diseases. In mice, and in human post-mortem studies, chronic social defeat stress (CSDS) and depression reduced histone H3 acetylation, and enhanced methylation of H3K27 in the regions close to *RAC1’s* promoter, causing decreased transcription of *RAC1* in the subcortical reward region of the *nucleus accumbens* (NAc). Downregulation of *RAC1* disrupted neurons actin dynamics, resulting in impaired synapsis and a depressed mice phenotype. *RAC1* knock down mice displayed antisocial behaviours, while *RAC1* overexpressing mice showed an anti-stress effect even after being subjected to bigger and stronger mice bullying, suggesting *RAC1* expression can help modulate depressive behaviours [145].

## Therapeutic perspectives

Until recently, small RHO GTPases including RAC1 were considered undruggable or, at best, difficult targets. To date, most attempts to inhibit RAC1 signalling have targeted either its localization (*e.g*., by attempting to impede its isoprenyation) using statins or GGTase inhibitors (GGTIs), or by blocking its effector, such as PAKs (Frax-1036), PI3Ks (TGX221, GSK2636771) or the MRTF/SRF complex (CCG203971) [108,136,137,150] (Figure 2). However, in the last decade, a number of small molecule inhibitors of RAC1 have been described, including NSC-23766, its derivatives MBQ-167and Ehop-016, as well as 1A–116, GYS32661, EHT1864, and AZA-1 and AZA-97 [150]. The mechanisms of action of RAC1 inhibitors falls into two main types: those that impede GTP binding and RAC1 interaction with downstream effectors, maintaining RAC1 in an inactive state, such as MLS000532223 and EHT1864 [151,152]; and those that interfere with GEF/RAC1 interactions such as NSC-23766 [153,154] (Figure 2). RAC1 inhibition by NSC23766 induced G_1_ cell cycle arrest and apoptosis in breast cancer cells and inhibited the growth of prostate PC-3 cancer cells. NSC-23766 has never been tested in a clinical setting due to its low potency and low tumour-targeting specificity [155,156]. Current assays using a polymeric nanoparticle drug delivery system loaded with NSC23766 have been shown to increase the drug concentration and retention in the tumour site in PC-3 bearing mice, resulting in a decrease in tumour growth without notable toxicity effects [102]. Inhibiting RAC1 with EHT1864 increased the effect of anti-breast cancer drugs in endocrine resistant breast cancer [74]. While these compounds continue to be studied in preclinical settings, at present there are, to our knowledge, no RAC1-specific inhibitors in clinical development.

The recent identification of direct inhibitors of RAS raise hope that similar approaches may yield small molecule inhibitors of RAC1. Recently, the Shokat lab has shown that, like KRAS, RAC1 contains a cryptic, highly dynamic pocket in the switch II region that is potentially druggable [157]. Using a RAC1^G12C, K96H^ mutant, they found that the KRAS^G12C^ inhibitors divarasib and MRTX1257 were able to bind RAC1, as well as RAC1^P29S^, and inhibit their ability to engage downstream effectors such as PAK1 and to propagate signals to the cytoskeleton in a dose dependent manner. While these proof-of-concept experiments were carried out with RAC1 mutations (*i.e*., RAC1^G12C^, alone or combined as RAC1^G12C, K96H^) that are rarely if ever encountered in human cancer, they point to the possibility of developing targeted inhibitors that act directly on cancer-relevant mutants such as RAC1^G12V^ and RAC1^P29S^ [157].

Finally, given the importance of maintaining a balance in RAC1 expression, transport, and signalling in cells, regulating epigenetic modifications of the *RAC1* gene could be useful as a therapeutic approach in cancer and other disorders.

## Conclusion

RAC1 is a signalling molecule that can be rapidly activated and deactivated, and that regulates a wide variety of processes in virtually all cells. RAC1 is a challenging therapeutic target, not only because of its structure, but because of its essentiality for many fundamental cell functions. For RAC1, as for many small GTPases, the challenge is to design new drugs to target specific mutations, the splicing mechanism, or to control downstream effector activity, without unduly disturbing the essential functions of WT RAC1. While past attempts to target RAC1 signalling have shown a modest degree of success, the potential of exploiting secondary pockets, as has been done for RAS and recently extended to RAC1, presents an intriguing opportunity for further drug development. Further explorations of *RAC1* epigenetic regulatory mechanisms might also suggest therapeutic opportunities. Finally, more work is needed to investigate how RAC1 expression and activity engenders immune escape mechanisms and affects immune cell populations.

## Data Availability

Data sharing is not applicable to this article as no new data were created or analysed in this study.
